# Occupational Risk Prevention through Smartwatches: Precision and Uncertainty Effects of the Built-In Accelerometer

**DOI:** 10.3390/s18113805

**Published:** 2018-11-06

**Authors:** Luis Sigcha, Ignacio Pavón, Pedro Arezes, Nélson Costa, Guillermo De Arcas, Juan Manuel López

**Affiliations:** 1 Instrumentation and Applied Acoustics Research Group (I2A2), ETSI Industriales, Universidad Politécnica de Madrid, Campus Sur UPM, Ctra. Valencia, Km 7., 28031 Madrid, Spain; luisfrancisco.sigcha@upm.es (L.S.); g.dearcas@upm.es (G.D.A.); juanmanuel.lopez@upm.es (J.M.L.); 2 ALGORITMI Research Center, School of Engineering, University of Minho, 4800-058 Guimaraes, Portugal; parezes@dps.uminho.pt (P.A.); ncosta@dps.uminho.pt (N.C.)

**Keywords:** wearable, risk assessment, smartwatches, microelectronic mechanical systems (MEMS) accelerometers

## Abstract

Wearable technology has had a significant growth in the last years; this is particularly true of smartwatches, due to their potential advantages and ease of use. These smart devices integrate sensors that can be potentially used within industrial settings and for several applications, such as safety, monitoring, and the identification of occupational risks. The accelerometer is one of the main sensors integrated into these devices. However, several studies have identified that sensors integrated into smart devices may present inaccuracies during data acquisition, which may influence the performance of their potential applications. This article presents an analysis from the metrological point of view to characterize the amplitude and frequency response of the integrated accelerometers in three currently available commercial smartwatches, and it also includes an analysis of the uncertainties associated with these measurements by adapting the procedures described in several International Organization for Standardization (ISO) standards. The results show that despite the technical limitations produced by the factory configuration, these devices can be used in various applications related to occupational risk assessment. Opportunities for improvement have also been identified, which will allow us to take advantage of this technology in several innovative applications within industrial settings and, in particular, for occupational health purposes.

## 1. Introduction

Wearables are electronic devices (or sensors) that can be used discreetly and comfortably on the body and, by using the embedded sensors, can be used to measure physical parameters such as movement, body temperature, and heart rate, among others. The sensors provide functionalities that are used in biometric and security applications. In recent years, there has been a large growth in this technological area along with the advances in telecommunications and mobile technology (mainly due to consumer electronics and miniaturization). Due to the diversity of sensors that can be integrated into smartwatches, they are currently used in many areas and applications such as health, wellness, safety, sports, fitness, communication, and business [1,2,3,4].

One of the most prominent wearable devices is the smartwatch. In recent years, these watches have been turned from portable mechanical devices used to measure time to electronic devices with features and functionalities similar to those available in smartphones, allowing the development of innovative applications in diverse areas. Smartwatches have advantages over smartphones, such as the location on the body and the direct contact with users’ skin, which facilitates the use of specific sensors such as a heart rate monitor, skin conductance and others [1,2,3,4,5,6].

Although the adoption of wearables is at an early stage, smartwatches have become the most popular wearables due to some of their advantages, such as their size, portability, and energy autonomy. Several studies have determined that the technological improvements, the development of innovative applications, and the reduction of costs can cause these devices to be massively adopted by the market [3,4,5,6,7,8,9,10,11].

Software applications for wearables devices are being constantly developed which take advantage of the increasing computer-processing capabilities, wireless communication, and different sensors. The sensors that are usually integrated into smart devices are accelerometers, gyroscopes, ambient light and magnetic sensors [1,2,12,13,14].

Currently, some researchers use specific wearable devices [15,16,17,18,19,20,21,22,23,24,25], smartphones with body adapters [26,27,28,29,30,31,32,33,34,35] and smartwatches [36,37,38] for developing occupational risk assessments. Wearables can be used for continuous monitoring and measurement over time, as well as warning or training devices for the worker; some of these applications take advantage of the wide range of available sensors and the discreet use of wearables, which makes them an excellent option for collecting information in an economic and discreet way [5,16].

In [39], certain applications of wearables have been identified for occupational risk assessment, among which are motion detection and physical activity, recognition of musculoskeletal injuries, fall detection, evaluation of exposure to physical agents, evaluation of exposure to chemical agents and the location of potential hazards (moving machinery). This work identifies that there is a growing number of solutions that are being proposed that are basically supported by the use of wearables. It is also mentioned that, in the future, this kind of system will take advantage of wireless communication capabilities to facilitate the exchange of information in real-time and to improve occupational safety management systems by using emerging technologies such as big data analysis.

With the current technical characteristics (processing speed, data storage, and wireless communication), wearable devices have the potential to be used as part of integrated risk management systems. The first experiences of integrated management systems based on wearable technology and the Internet of Things (IoT) for the industry are described in [22,40,41]. In [40], a framework has been established for the creation of intelligent work environments that allow risk assessment in a personalized manner in real-time using personal protective equipment with IoT technology, whereas in [22,41] industrial security management systems have been implemented that use wearables and IoT devices to supervise, raise awareness and send emergency alerts to workers to promote better labor practices and safe work environments. These studies show that the IoT approach can be used in sensors, tools, and workers using wearables for constant monitoring and real-time alerting. All this technology will allow the improvement of the management of occupational safety.

Based on the literature review, several applications have been identified in which the wearables’ vibration sensors are used (directly or indirectly) for occupational risk prevention (ORP), health, and safety. The identified applications are the evaluation of physical activity (PA), fall detection and vibration monitoring [42].

One of the main applications of wearables devices related to users’ health is the evaluation of PA. In [43], a bibliography referring to the development of the systems dedicated to movement detection is compiled. In these systems, triaxial accelerometers and movement classification methods based on threshold or statistical classification schemes are mainly used (supervised machine learning techniques). In this study, the typical applications of PA detection are also identified, including posture and movement classification, the estimation of the expended energy, and automatic fall detection.

In [44], a compilation of the techniques used to perform the monitoring of occupational physical activity has been made, in which sensors such as pedometers, accelerometers and global positioning systems (GPS) can be used simultaneously as integrated systems for the determination of physical activity in the workspace. This article concludes that the addition of sensors allows us to obtain physiological variables that can be used in other areas of occupational health.

Meanwhile, in [45], the effects produced by the location of accelerometers on the wrist or ankle for the estimation of PA in activities such as walking or cycling has been analyzed. The results show high precision in identification using accelerometers with a sample a rate of 90 Hz and configured to a range of ±4 g, using the support vector machine (SVM) classification algorithm. This study also mentions that it is possible to detect activities by using characteristics of the signal in the time or frequency domain, and it is feasible to decrease the sampling rate of the signal at 40 Hz without decreasing the accuracy of the classification algorithm too much.

Recent solutions have started to use commercial smartwatches to identify human activities. In [46], it is identified that a smartwatch’s accelerometer can provide accurate information to perform a satisfactory recognition of activities. This article shows that the technical requirements for the sensors depend on the application for which they are intended (e.g., monitoring of activity or inactivity, recognition of gestures or industrial monitoring related to exposure to vibrations), whereas in [47] it has been shown that it is possible to identify postures in an accurate way using low sampling rates in the accelerometer of a smartwatch, which can help to improve the battery life of the devices.

The different efficiencies in the recognition of activities between a smartwatch and a smartphone were compared in [48]: the results suggest that smartwatches can identify manual activities with a higher accuracy than smartphones, giving them the potential to be used in the biomedical and health domains. However, in [49], a smartphone and a commercial smartwatch were simultaneously used, showing that the combination of devices can increase the accuracy of the identification of activities. In addition, in [36], a smartwatch was used to identify gestures and activities, in addition to presenting information in a discreet way to the workers, through the screen of the device and haptic feedback. The developed system allows permanent assistance to the user during the performance of their work tasks. This article also presents new forms of interaction between the devices and the users, using novel applications in areas such as industry (mechanical and construction), the consumer market at home, and ambient assisted living.

Automatic fall detection is another application in which the wearables’ accelerometers have been extensively used. In [50], a compilatory study was made of fall detection and prevention systems using environmental and wearable sensors. The study determined that wearables have advantages over environmental sensor systems, such as better mobility, extended spatial coverage, easier installation and use. In [51], a complete systematic analysis of fall detection systems implemented in the Android platform was carried out. The results suggest that these devices can be a viable solution for fall detection due to their excellent cost-effectiveness ratio. Some open issues such as the lack of precision in the sensors and the reduced range in which the sensors are programmed by default were also identified; these issues reduce the attractiveness of this technology.

For work-related fall detection, a system is presented in [52] that uses various accelerometers coupled with safety equipment (belt and vest) placed on the chest, hip, arm, and hand. These sensors communicate with a smartphone and a server to predict potential falls through the automatic detection of fall precursor events with different classifying algorithms. The obtained results show a considerable accuracy in fall prediction by detecting precursor events. The authors have also concluded that it is possible to improve the accuracy of the prediction by increasing, for example, the number of used sensors.

The authors of [53] showed that it is possible to identify daily activities and detect falls with a smartwatch placed on the left hand using different pattern classification algorithms. In this article, it is highlighted that the position of the device on the wrist can decrease system performance due to random movements of the hand. In [54] a commercial smartphone and smartwatch with algorithms based on threshold analysis have been used in combination, demonstrating that the performance of the system to discriminate daily activities and falls can be increased with a small decrease in the efficiency in the detection of falls when performing simultaneous analyzes.

Solutions that use commercial smartwatches for the evaluation of risks associated with vibrations that affect the hand–arm system (HAV) have been also developed. An approach based on machine learning was presented in [37]. In this study, a system was developed which uses the built-in microphone and accelerometer of a commercial (not modified) smartwatch to recognize the kind of tool used and the time of use to estimate the value of the daily dose of vibration, *A(8)*. The results suggest that it is feasible to implement recognition systems for work tasks and use this information to prevent risks. In [38], a vibration measurement system was developed using the built-in accelerometer from a smartwatch to measure the value of *A(8)*, and it was concluded that this system can be used in specific HAV evaluation tasks due to the technical limitations imposed by the operating system on the sensors to increase the battery’s autonomy. However, the opportunities for improvement in the future and the smartwatches’ advantages, such as being a low-cost solution for the continuous monitoring of workers, its portability and comfort of use were highlighted, as well as the compliance with the principle of not increasing or causing new risks.

### Accelerometers

One of the primary sensors included in smart devices is the accelerometer. At present, this type of sensor is widely used as a stand-alone device, in smart devices or within wearable devices for specific applications such as the monitoring of physical and sports activities, fall detection, and the analysis of structural or machine vibrations, as well as vibrations that affect the human body [16,17,18,19,20,22,23,24,25,26,27,28,29,30,31,32,36,37,38].

Accelerometers are a type of inertial sensor that can measure acceleration in their sensitive axes. The principle of operation of the accelerometers is based on a mechanical sensor element, composed of a test mass attached to a reference frame with a mechanical suspension system. Based on this principle, the acceleration can be measured electrically, using the physical changes in the displacement of the test mass. There are three types of accelerometers: piezoresistive, piezoelectric and capacitive [44,55].

The accelerometers used in smartwatches are called MEMS (microelectronic mechanical systems): these sensors have a reduced dimension and can be used to capture different physical variables [1]. The technical characteristics and performance of MEMS accelerometers have been analyzed in several studies, in most cases comparing them with piezoelectric accelerometers (PCB). The results of these studies concluded that these sensors are a viable and low-cost solution that can be used in different applications [56,57,58,59,60,61,62,63,64].

In the work of Tarabini [65], the technical characteristics of the MEMS accelerometers and their possibilities for use in the evaluation of hand–arm vibrations, full body and other types of mechanical vibration measurement have been evaluated. This article shows that this kind of transducer complies with most of the technical requirements of ISO 8041:2005 [66]. The limitations found in the frequency response can be improved using equalization. Therefore, these sensors present advantages over PCB, such as their reduced cost and size, which allow the sensor to be used directly on the handles of tools or fixed on the skin.

Although there is an extensive bibliography focused on the analysis of the different characteristics of MEMS accelerometers, there are fewer studies that analyze the behavior of sensors within the wearable device (specific device or smartphone). To the best of our knowledge, no study has been identified that specifically analyzes the performance of accelerometers within commercial smartwatches.

The MEMS accelerometers included in the device circuitry have enough technical characteristics for optimal operation in specific applications [39]. However, when they are used in intelligent devices, their performance is reduced by the operating systems to reduce the energy consumption and increase the energy autonomy. Even with this reduction, intelligent devices can be used in many applications. Knowing the behavior and limitations of the accelerometer (which is one of the most commonly used sensors) can be useful in future research in which the use (direct or indirect) of the accelerometers in a commercial smartwatch is considered.

The main objective of the current paper is to determine the accuracy and uncertainty associated with the acceleration measurements made by the vibration sensors of a few current commercial smartwatches. The results are used to analyze the current and future potential applications related to the prevention and management of occupational risks. It is also discussed whether these devices can be used as precision measuring instruments, control instruments or as training and teaching devices.

Specifically, this paper presents the methodology used for the characterization of the frequency response and variability of the amplitude of the acceleration magnitude captured by the MEMS accelerometers of three commercial smartwatches from different manufacturers. The smartwatches used in the experiments use the Android Wear operating system (version 1.5, Google), which facilitates the preparation of trials and the comparison of results.

## 2. Materials and Methods

The amplitude and frequency response of three commercial smartwatches was evaluated in order to analyze the accuracy of the accelerometer measurements. In this analysis, the repeatability of the measurements has been considered, and the associated uncertainties have been calculated.

In this kind of intelligent device, it is possible to obtain the digital representation of the acceleration signal in the three axial axes from the sensor (triaxial MEMS type accelerometer) to analyze its amplitude and frequency. The amplitude can be analyzed by using the maximum amplitude (peak value) of the signal or by analyzing the root mean square value (RMS), while the frequency tests can be carried out using discrete signals such as sinusoidal waves at different frequencies. Also, the amplitude and frequency test can be performed in the frequency domain by using the Fourier transform with test signals such as pink noise or frequency sweeps of sine waves [67].

In the current study, the amplitude and frequency analysis is performed by analyzing the RMS values of sinusoidal vibration signals at discrete frequencies. The mechanical signals used in the measurements were generated by a vibration shaker.

Also, the estimation of the maximum sampling rate of each of the smartwatches’ accelerometers was verified at first by using time-stamps created during the capture of signals. It has been identified that the sampling time-intervals of the accelerometer are not constant, which generates jitter errors. The average sampling rates of the smartwatches configured in Android Studio 3.2 (Google, California, USA) at the fastest sensor sampling rate are 250 Hz for smartwatch 1, 200 Hz for smartwatch 2, and 50 Hz for smartwatch 3.

The analysis was made at different amplitudes to verify the amplitude and frequency behavior of the built-in accelerometers. The analysis methodology was chosen according to the existing bibliography and ISO standards regarding the measurement instruments used for the analysis of the human response to vibrations [66] and secondary accelerometer calibration methods [68].

This section has been divided into six subsections. Section 2.1 describes the technical characteristics of the built-in accelerometers from the smartwatches used in the tests. Section 2.2 describes the test bench used in the calibration and measurement tests. Section 2.3 details the development of the vibration measurement system for the Android test devices. Section 2.4 presents the process of calibration and adjustment of the implemented measurement system in the devices, before performing frequency and amplitude tests. Section 2.5 describes the frequency response measuring process of the test devices at different amplitudes. Lastly, Section 2.6 presents the procedure used to perform the analysis of uncertainties associated with calibration and measurements.

### 2.1. Test Devices

Three commercial smartwatches with the Android Wear system (version 1.5, Google) were used to perform the amplitude and frequency analysis. For the choices of the test smartwatches, several high-end commercial smartwatches available on the market in 2017 were identified.

From these devices, two smartwatches were chosen which had the highest factory-set sampling rate in the Android Wear system for the accelerometer. In addition, a third smartwatch was chosen with a standard sample rate factory-set for movement analysis to verify its behavior.

The three smartwatches have similar technical characteristics, and their sensors are programmable. However, the factory settings (maximum sampling frequency and amplitude range) for the Android system are lower than that at which accelerometers can work.

The technical characteristics of the smartwatches and the built-in accelerometers are specified in Table 1.

### 2.2. Test Bench

**Figure 1:**
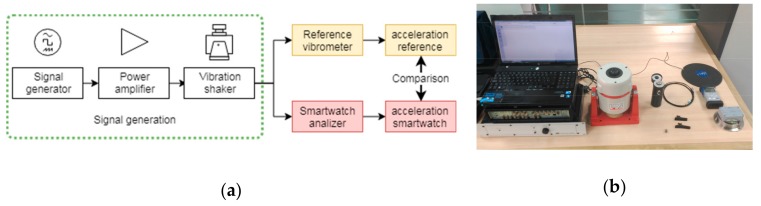
Vibration analysis and generation system: (**a**) block diagram of the connections; (**b**) devices of the test bench.

The calibration processes and the amplitude and frequency measurements were made by using a vibration generation and analysis system composed of a PULSE 7537 system (Bruel & Kjaer, Copenhagen, Denmark), connected to an LDS PA 100E power amplifier (Bruel & Kjaer), which feeds to an LDS V406 CE M4 vibration shaker (Bruel & Kjaer). A SV106 vibrometer (Svantek, Warsaw, Poland) connected to a Dytran 3023M3 accelerometer (Dytran Instruments, California, USA) was used as a reference. The equipment was connected according to Figure 1.

### 2.3. Development of the Vibration Measurement System for the Android Wear Devices

**Figure 2:**
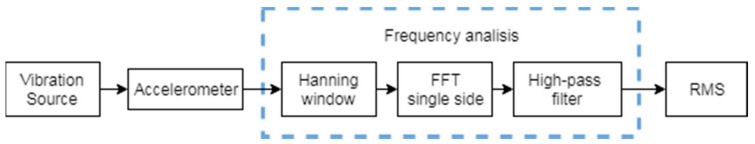
Vibration measurement system (frequency domain analysis). FFT, fast Fourier transform; RMS, root mean square value.

A software application for Android Wear devices was developed [69] and installed in the smartwatches to determine the RMS acceleration value. The application captures and processes the triaxial accelerometer signals at the maximum frequency sample rate allowed by the device. The application shows, as a result, the RMS acceleration values without the gravity offset. The scheme in Figure 2 was used to implement a measurement system following the guidelines of ISO 8041:2005 [66].

The acceleration analysis for each of the three axial axes was made in the frequency domain using the fast Fourier transform (FFT) [70]. The digital signals captured from the sensor must be subjected to the windowing process before the transformation to the frequency domain; after that, the signal must be filtered to avoid the effects of gravity using a high-pass filter that eliminates low-frequency components.

The RMS (total band power) value for each analysis window is obtained using the linear integration method, calculated using the Parseval theorem described in the following equation:

(1) $$\mathrm{RMS}=\sqrt{2\sum_{f=0}^{f=SR/2}{\left|X\left(f\right)\right|}^{2}},$$

where |*X*(*f*)| is the module of the FFT components in each spectral line.

The values displayed by the smartwatch measurement system are obtained from the moving average of the RMS acceleration, which is calculated using the instantaneous values of the acceleration of each analysis window.

### 2.4. Calibration and Adjustment through the Comparison Method

Although the smartwatches have a factory-calibrated triaxial accelerometer, calibration and adjustment tests must be performed before performing frequency and amplitude measurement tests on the devices. The calibration tests must verify the degree of deviation of the measurements obtained with the smartwatch to later perform the adjustment process and compensate this deviation to avoid mistakes in data collection in the rest of the experiments.

The calibration process was made by adapting the guidelines of ISO 16063-21:2003 [68] regarding the calibration of a vibration transducer by comparison to a reference transducer. From the different methods of secondary calibration that exist, the method of comparison with a reference accelerometer is the most used due to its ease of implementation and reliability. The method consists of coupling the accelerometer to be calibrated on another accelerometer which is pattern calibrated and then driving the coupled pair with vibrations with a determined frequency and amplitude.

For this study, the calibration of the vibration measurement system in smartwatches consists in making a comparison between the smartwatch acceleration measurement value and the reference to get the acceleration bias and its associated uncertainty. For this process, the reference accelerometer and the smartwatches were coupled using adhesive. For smartwatches 2 and 3, it was necessary to use a thin plate between the devices due to the size and the straps of the smartwatches.

The calibration of the devices can be performed at any frequency and amplitude within the operational limits of the device. The ISO 16063-21:2003 [68] standard recommends using the standardized third-octave frequencies indicated in ISO 266:1997 [71] and amplitudes of 1, 2, 5 or multiples of 10 m/s^2^.

In the case of digital devices, since it is not possible to access the voltage output of the accelerometer directly to get the sensitivity value, the calibration can be performed by verifying the measurement error, which is the difference between the acceleration value obtained with the test device and the value of the reference device. By getting the value of this error, a correction factor can be applied to each accelerometer axis to get the RMS value. For the calibration of the smartwatches, a mechanical sinusoidal vibration signal with an amplitude of 10 m/s^2^ at a frequency of 12.5 Hz was used, considering the limitations of the devices in both amplitude and frequency.

### 2.5. Test of the Frequency Response at Different Amplitudes

The present test was made to verify the smartwatches’ frequency linearity at three different amplitudes. The test was made following the guidelines of the ISO 8041:2005 [66] standard and the ISO 16063-21 [68] criteria for coupling the reference and the test transducers.

According to ISO 8041:2005 [66], when performing measurements at different amplitudes and frequencies, the indication error (*Ex*) can be expressed in absolute terms with the difference between the value of the analyzed device and the value shown by reference analyzer (Equation (2)). The ISO standard also explains that the indication error can be shown in relative terms, expressed in decibels or the percentage of deviation from the reference value. The deviation percentage (*ε*) for the reference can be calculated using Equation (3) [66].

(2) $$Ex=a_{test}-a_{ref},$$

(3) $$\varepsilon =\left(\frac{a_{test}-a_{ref}}{a_{ref}}\right)*100\%.$$

The frequency response tests were performed by adapting the method of comparison with a reference accelerometer described in ISO 16063-21 [68] (in a similar way to the calibration test). The transducers were coupled according to the procedures described in the method of calibration of accelerometers by comparison (back-to-back calibration method). The results of these measurements are the RMS acceleration values of the deviation percentage to the reference vibrometer and its associated uncertainties at different frequencies and amplitudes.

The mechanical test signals were generated with the vibration shaker fed by discrete sinusoidal waves at third-octave intervals, with central frequencies in the range of 6.3–100 Hz. The frequencies were selected based on the maximum frequency analysis of each device according to Nyquist’s theorem [67]. The vibration amplitudes used in the test were 10, 5 and 2 m/s^2^ RMS.

The reference accelerometer and each smartwatch were simultaneously coupled to the shaker’s vibrating surface; then, six RMS acceleration determinations were performed at each of the frequencies, keeping the acceleration amplitude constant, then repeating the process for the other amplitudes mentioned.

### 2.6. Calibration and Measurement Uncertainties

Due to the sources that can produce variability in the measurements of the magnitude of vibration, such as the physical equipment conditions, experimental variability, and environmental factors such as humidity, temperature or atmospheric pressure, it is necessary to perform the analysis of uncertainties associated to the measurements and the calibration to be able to characterize the dispersion of the values that could reasonably be attributed to the measurand.

The estimation and expression of the uncertainties of the calibration and measurement results were made following the criteria and guidelines of ISO/IEC Guide to the Expression of Uncertainty in Measurement (GUM) [72] and adapting ISO 16063-21:2003 [68]. Significant contributions to the final uncertainty are considered as each of the corrections of the devices used in the calibration and measurement processes:

- The uncertainty associated to the reference device.
- The temporary drift of the reference device.
- The scale division of the reference device and the device to be calibrated.
- The repeatability of the test during the calibration process.
- The alignment of the device to be calibrated against the reference.
- Environmental effects (temperature and humidity variation).
- Variations produced by the magnetic field of the vibration shaker.
- The uncertainty of the power amplifier and the vibration shaker.
- The uncertainty produced by the sampling rate variation of the smartwatches.

#### 2.6.1. Model

According to ISO-GUM, for the determination of a measurand, other quantities that are related through a functional relationship are normally used. The function contains all quantities, including the corrections and correction factors for systematic effects that can bring a significant component of uncertainty to the measurement result. The corrections and correction factors are applied to the model to compensate for the significant influences that affect the measurement results due to the effects of systematic and random errors [68,72].

For the determination of uncertainty associated with calibration and measurements, it is necessary to adapt the procedure described in ISO 16063-21:2003 [68], because the output voltage of the accelerometer to be calibrated cannot be directly accessed to use the sensitivity as a function to calculate the uncertainty.

In the current case, to facilitate the uncertainty calculations of the calibration and measurements, the indication error (*Ex*) shown in Equation (2) has been used as a function of the model (Equation (4)). In this model, the corrections produced by systematic effects are considered.

(4) $$Ex=\left(a_{test}+c_{temp\;test}+c_{res\;test}+c_{calib\;test}+c_{cross\;test}+c_{jitt\;test}\right)-\left(a_{ref}+c_{temp\;ref}+c_{res\;ref}+c_{calib\;ref}+c_{cross\;ref}\right),$$

where:

- *c_temp_* is the correction due to the temperature;
- *c_res_* is the correction due to the finite resolution of the meter;
- *c_calib_* is the correction due to the calibration of the meter;
- *c_cross_* is the correction due to the cross sensitivity;
- *c_jitt_* is the correction due the sampling rate variation in the smartwatch (jitter error).

The components of uncertainty have been expressed as relative standard uncertainties to simplify the calculations, as mentioned in [73]. The relative uncertainty values have been calculated using Equation (3).

Although in some cases the value of the corrections can be a null value when calculating the indication error, their uncertainties will not be null. The coefficients of sensitivity for each contribution are equal to one because the input quantities can be considered as uncorrelated for the calculation of the relative expanded uncertainty, as indicated in [68,73].

#### 2.6.2. Characterization and Estimation of the Value of Contributions Type A and Type B

According to GUM [72], the uncertainty components can be grouped into two categories according to the evaluation method: “type A” and “type B”. The classification into type A and type B consists of two different ways of evaluating uncertainty components, based on probability distributions.

The characterization of the sources of uncertainty used in the analysis are described in Table 2. Some sources of uncertainty indicated in ISO 16063-21:2003 [68] have been ignored because they are not components of the proposed model, or their information is not available.

For the estimation of the combined uncertainty, all the sources of uncertainty from the proposed model have been analyzed. The uncertainty associated to the acceleration of the meters has been analyzed as a normal distribution because this variable is determined by performing several quantity determinations.

The uncertainty components of the temperature, the finite resolution of the meter and the cross-axis sensitivity have been analyzed as rectangular distributions because only the range in which the quantity can be found is known.

The uncertainty contribution of the devices’ calibration is analyzed as a normal distribution. Also, the uncertainty contribution of the sampling rate of the smartwatch has been analyzed by using the Monte Carlo method [74] with the model indicated in [75].

The combined relative standard uncertainty associated to the calibration test is estimated using Equation (5), according to the law of propagation of the variances. For uncorrelated elements, the relative combined uncertainty can be estimated by Equation (6), adapted from ISO 16063-21:2003 [68].

(5) $$u_{c,rel}\left(y\right)=\sqrt{\sum_{i=0}{\left(\frac{\partial f}{\partial x_{i}}\right)}^{2}u_{rel\;i}{}^{2}\left(x_{i}\right)+2\sum_{i=0}^{N-1}\sum_{i=0}^{N}\frac{\partial f}{\partial x_{i}}\frac{\partial f}{\partial x_{j}}u_{rel}\left(x_{i},x_{j}\right)},$$

where:

- *u_c,rel_* is the combined relative standard uncertainty;
- *y* is the measurand determinate for *N* input quantities *x*_1_, *x*_2_, …, *x_n_*;
- *∂f/∂x_i_* are partial derivatives evaluated at *X_i_* = *x_i_*;
- *x_i_* are estimated input values;
- *u_rel_* (*x_i_*, *x_j_*) are estimated covariance.

(6) $$u_{c,rel}^{2}\left(y\right)=\overset{n}{\underset{i=0}{\sum }}{\left(\frac{u_{rel\;i}c_{i}}{x_{i}}\right)}^{2},$$

where *c_i_* are sensitivity coefficients.

As indicated in [68], the results of the measurement must be reported as the expanded uncertainty. In this case, it is necessary to multiply the value of the combined uncertainty by a coverage factor *k* to get the expanded uncertainty. As mentioned in the ISO 16063-21:2003 standard, it has been considered that the results of the measurements follow a normal distribution so that the coverage factor *k* = 2 can be used for coverage of approximately 95%. The expanded relative uncertainty can be estimated by Equation (7), adapted from [68].

(7) $$U_{rel}\left(y\right)=ku_{c,rel}\left(y\right).$$

The same model and method were used to calculate each of the uncertainties associated with the frequency response measurement tests, obtaining results for each of the analysis points. The calculation of the associated uncertainties was performed using the relative uncertainties expressed as a percentage to facilitate the calculation and expression of results.

## 3. Results

The following section shows the results achieved in the experiments of the frequency amplitude response of the three test smartwatches. In Section 3.1, the results of frequency linearity are illustrated graphically at different amplitudes. Section 3.2 details the smartwatches’ measurements of deviation as compared to reference vibrometer measurements, expressed in percentages, together with their respective associated uncertainty.

### 3.1. Frequency Response Test at Different Amplitudes

**Figure 3:**
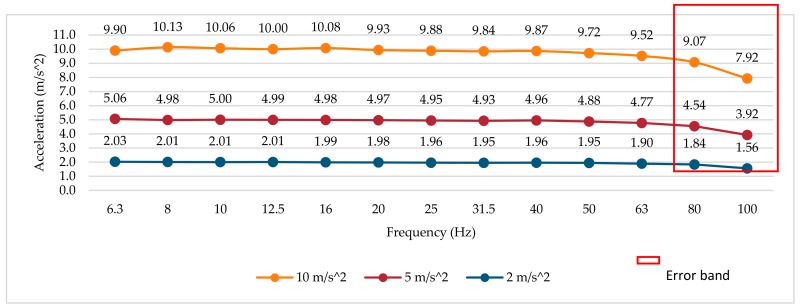
Smartwatch 1 frequency response for three amplitudes.

**Figure 4:**
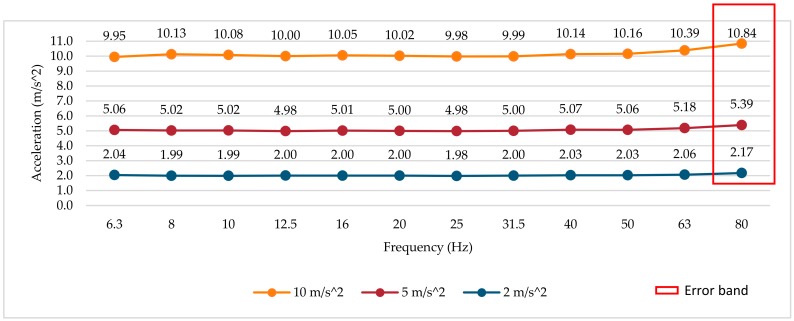
Smartwatch 2 frequency response for several amplitudes.

**Figure 5:**
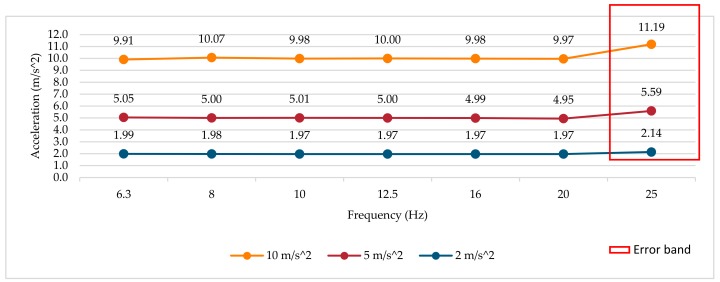
Smartwatch 3 frequency response for several amplitudes.

The results of the tests were obtained from the measurements of the RMS magnitude of the three smartwatches (average value of six determinations at each point) in the z-axis according to the Android sensor coordinate system [76]. The reference vibrometer is used to adjust the amplitude value of the mechanical test signal to the different test frequencies and amplitudes. The frequency analysis is performed only for the first third-octave bands, due to the maximum sampling frequency of each smartwatch. The frequency response curves in three different amplitudes (10, 5 and 2 m/s^2^) are shown in Figure 3, Figure 4 and Figure 5, respectively. An error band has been defined for amplitude and frequency linearity according to the specifications of ISO 8041:2005 [66], where values with a deviation greater than 6% are considered as amplitude linearity errors. Deviation values greater than 6% with respect to the reference have been highlighted in the figures and tables.

#### 3.1.1. Smartwatch 1

The frequency response curves of smartwatch 1 (see Figure 3) show a relatively flat frequency response up to the frequency of 40 Hz for the three amplitudes. For frequencies higher than 50 Hz, the amplitude gradually decreases; this fall in the amplitude could be produced by the slope of the anti-aliasing filter applied by the sensor before the digitalization and the fixing method. A deviation of more than 6% (error band) has been identified in the bands of 80 and 100 Hz.

#### 3.1.2. Smartwatch 2

For smartwatch 2 (see Figure 4), the frequency response curves present a relatively flat response up to the frequency of 50 Hz for the three vibration amplitudes used in the tests. For frequencies higher than 63 Hz, the error increases up to a maximum of 0.84 m/s^2^. This increase in amplitude at high frequency could be produced by the physical location of the accelerometer inside the device, the accelerometer and smartwatch fixing method (different from smartwatch 1, due to its size) and the vibration modes of the device itself. In this smartwatch, the deviation induced by the anti-aliasing filter is not noticeable; this may be due to the cutoff frequency of the low pass filter, which can be greater than 100 Hz. A deviation of more than 6% has been identified in the band of 80 Hz.

#### 3.1.3. Smartwatch 3

For smartwatch 3 (see Figure 5), the frequency response curves have a relative flat response up to the frequency of 20 Hz for the three amplitudes analyzed. For the maximum frequency analyzed of 25 Hz, the error increases up to 1.19 m/s^2^; this increase in the amplitude can be produced by the sampling error (jitter), the accelerometer and smartwatch fixing method (different from the fist smartwatch, due the difficulty of removing the smartwatch’s straps) and the location of the sensor and the vibration modes of the device itself. The frequency analysis in this device has only been possible in the first seven third-octave bands because the maximum sampling frequency set at the factory is 50 Hz.

As in smartwatch 2, there is no decay in the level at frequencies of 20 or 25 Hz induced by the anti-aliasing filter. A deviation of more than 6% has been identified in the band of 25 Hz.

### 3.2. Results of the Expanded Relative Uncertainties and Relative Deviation of the Smartwatches Measured from the Reference at Different Amplitudes and Frequencies

In order to compare the deviation of the measurements at different amplitudes of each smartwatch, Table 3, Table 4 and Table 5 show the relative deviations expressed as the deviation percentage (*ε*) and the expanded relative uncertainty (*U_rel_*) (expressed in percentage) of each smartwatch with respect to the reference vibrometer at each frequency and amplitude analyzed in the previous test. The deviation percentage (*ε*) values are determined using Equation (3).

The uncertainty values from each of the frequencies and amplitudes were calculated through the calibration model by using the comparison method for all the frequencies and amplitudes in the three devices.

#### 3.2.1. Smartwatch 1

Although the absolute error of the device at high frequencies is different according to the magnitude of the vibration signal (see Figure 3), the relative error expressed in percentage for each frequency band at different amplitudes is similar, reaching a maximum of −20.82% at the frequency 100 Hz (see Table 3). These results show a linear behavior with respect to the amplitude in the entire analysis range of the device. The deviation in the error band of 6% is exceeded in the bands of 80 and 100 Hz.

#### 3.2.2. Smartwatch 2

For smartwatch 2, the relative error expressed in percentage remains at low values (see Table 4). For frequencies higher than 63 Hz, the error increases up to a maximum of 8.41%. This increase in the frequency response at high frequencies could be produced by the physical location of the accelerometer inside the device and the vibration modes of the device itself. In this smartwatch, the errors induced by the anti-aliasing filter are not noticeable. The deviation in the error band is exceeded in the band of 80 Hz.

#### 3.2.3. Smartwatch 3

For smartwatch 3, the relative error expressed in percentage remains at low values until 20 Hz (see Table 5). For frequencies higher than 25 Hz, the error increases up to a maximum of 11.9%. As with smartwatch 2, the increase in amplitude at high frequencies could be produced by the physical location of the accelerometer inside the device and the vibration modes of the device itself. Errors induced by the anti-aliasing filter are not noticeable. The deviation in the error band is exceeded only in the band of 25 Hz.

In the three devices, a gradual increase in the uncertainty can be observed when increasing the frequency of analysis. This behavior is produced by the contribution of uncertainty produced by the variation in the sampling rate (jitter effect) that affects the high frequencies that are close to the Nyquist frequency.

## 4. Discussion

The frequency analysis of the three devices can only be performed for the first third-octave bands, depending on the maximum sampling rate of each smartwatch. The limitation identified in the bandwidth restricts the use of these devices to specific evaluation tasks related to occupational risk prevention.

The value of 10 m/s^2^ RMS was used as the maximum amplitude of analysis, because higher amplitudes vibrations can saturate the accelerometer depending on its orientation with respect to the ground due to the gravity offset. The acceleration magnitude added to the gravity offset can exceed the factory set amplitude limit of ±2 g (13.85 m/s^2^ RMS) easily.

### 4.1. Analysis of the Frequency Response at Different Amplitudes

Regarding the frequency response analysis, smartwatch 1 presents the higher analysis bandwidth (125 Hz), and presents a flat frequency response up to the frequency of 40 Hz with a maximum amplitude range of ±2 g. This characteristic makes this device an excellent option for vibration analysis tasks that require a range up to the band of 80 Hz.

For smartwatch 2, the flat frequency response makes this device an excellent option to perform vibration measurements. The primary considerations for this device are the maximum range of frequency analysis, due to the sampling rate (200 Hz) and the amplitude range (±2 g) configured at the factory.

For smartwatch 3, the results present a flat frequency response at low frequencies. However, due to its reduced sampling rate (50 Hz) and its amplitude range (±2 g), this device can underestimate the magnitude of vibrations when performing vibration measurement tests with signals that have frequencies higher than 25 Hz.

### 4.2. Measurement Uncertainties Analysis

The values obtained in the test of amplitude and frequency are in the range from ±3.0% to ±3.8%. According to ISO 8041:2005, the expanded uncertainties for mechanical frequency response tests should be a maximum of 4.5% for pattern device verification and 5% in periodic verification for users. These maximum values have not been exceeded in any of the tests, even at very high or low frequencies where it is possible to get variability in the test signal due to the technical characteristics of the equipment.

In this analysis, the uncertainty associated with the calibration of the reference vibrometer has been identified as the primary source of contribution to the combined uncertainty, followed by the contribution to the uncertainty produced by the finite resolution of the meters, especially when analyzing low acceleration amplitudes (5 and 1 m/s^2^).

A gradual increase in the uncertainty has been noticed in the three devices when increasing the frequency of analysis. The increase in the uncertainty is produced by the jitter effect due to the variation in the sampling rate [75].

The other uncertainties sources analyzed have been characterized by having a low contribution to uncertainty because the tests were conducted in a laboratory with a controlled environment.

### 4.3. Possible Uses with the Current Configuration and Limitations

Although the smartwatches that use the Android Wear system have certain limitations in bandwidth and the maximum amplitude analyzed with the accelerometer, several applications for occupational risk assessment have been identified in which the vibration sensor can be used with the current factory configuration.

In addition, in the bibliography review, two main limitations for the identification of activities and the analysis of hand–arm vibrations have been identified which are related with the location of the device and the sensor in the body. The first limitation is that the smartwatch is located on the wrist and there can be a relative movement between the sensor itself and the bone, due to the presence of soft tissues that can make the analysis of the vibration signals difficult.

The second limitation regards hand–arm vibration assessment. In this case, the sensor must be placed between the part of the body exposed to vibrations and the vibrating surface; therefore, when evaluating the magnitude of vibrations with smartwatches, changes in the acceleration response can occur because the transmissibility between the palm and the wrist is not in unity [77].

#### 4.3.1. Smartwatches for Physical Activity

For monitoring occupational physical activity, it has been identified that the technical characteristics of the smartwatches analyzed in this article can meet the sampling rate requirements in activities such as sleep detection, heart rate [47], ambulatory movement (frequencies of analysis in the range of 1–3 Hz) [44], identification of postures (sampling rate of 10 Hz) [48], identification of specialized manual activities (sampling rate 20 Hz) [49], and for environmental interaction in the work environment using gestures (25 Hz sampling rate) [36]. In these applications, low requirements in the sampling rate are identified, while to identify the PA with sensors placed on the wrist in [46], it is mentioned that it is possible to use a sampling frequency of 40 Hz without degrading the accuracy of the classifier.

#### 4.3.2. Smartwatches for Fall Detection

For fall detection, different results are identified depending on the configuration of the devices. In [78], it is mentioned that for fall detection using smartphones, the acceptable amplitude ranges of the devices are ±4 or ±16 g. This range is higher than the one currently configured in the factory (±2 g). In [79], a 100 Hz sampling frequency has been used in a smartphone, getting satisfying results in the fall detection.

In [53], the authors suggest that the current factory configuration of Android devices may not have the necessary precision due to the built-in accelerometer sample rate. The operating system allows the configuration of the sample rate in a range from 7–200 Hz depending on the accelerometer model. Also, the sample rate presents some variation over time. Besides this, it is mentioned by other authors such as Abbate and Fudicar [80,81] that using low sampling frequencies (50 Hz) is a good compromise between performance and energy consumption in the fall detection.

For fall detection with wearables placed on the wrists, in [54], fall datasets have been recorded and evaluated using commercial devices with inertial sensors. The maximum sample rate of this devices was 50 Hz; with this configuration, a high percentage of precision in fall detection has been achieved. These results suggest that is possible to get reliable performance in fall detection using the current accelerometer factory configuration in smartwatches, also maintaining a useful energy autonomy of the device to be used continuously over time. 

#### 4.3.3. Smartwatches for Hand–Arm Vibration

For the measurement of the magnitude of the vibrations in applications related to exposure to hand–arm vibrations, the results show that the current configuration of the Android smartwatches does not meet the requirements for accurate measurement in the complete range of frequency and amplitudes [38].

When comparing the results of the smartwatches’ measurements with the results achieved by Tarabini (direct analysis of the MEMS accelerometers), it is confirmed that the main limitations are the reduced bandwidth and the lack of linearity found in the high frequencies, although, depending on the application, the linearity problems can be overcome by sensor equalization.

The analyzed accelerometer factory configuration of the smartwatches (in amplitude and frequency) is lower than the described by Tarabini [65]. However, it is feasible to use some smartwatch models for the evaluation of vibrations that affect the whole body. Because the analysis bandwidth proposed by ISO 8041:2005 is from 0.5–80 Hz for health and well-being. On the other hand, for hand–arm vibrations, the current configuration of the smartwatch can make mistakes in the measurement of the vibration magnitude when analyzing vibrations with high magnitudes and extended frequency content.

As described by Moschioni [82], the vibration direct measurement is the best procedure for assessing the hand–arm vibration-related risk with minimum uncertainty; nevertheless, to perform the hand–arm vibration risk assessment with the actual sensor configuration, it could be convenient to use accelerometers for the detection of a pattern of activities and tools and then estimate the vibration exposure instead of directly measuring the magnitude, as proposed in [37].

The sensor configuration problem can be solved quickly with improvements in the energy consumption management of the devices and sensors, and, when the wearables APIs allow, a more detailed configuration of the characteristics of the accelerometers.

In the work of Matthies [37] referring to the estimation of the exposure to vibrations using an Android smartwatch with an accelerometer configured with a sampling frequency of 50 Hz, it has been identified that smartwatches have a sufficient precision to analyze and detect several kinds of tools. However, with the simultaneous use of a smartwatch’s microphone, 85% of the overall detection rate has been achieved in several tools. Thus, with the results achieved in this study for the smartwatches, it is feasible to get an improvement in the percentage of accuracy in the detection of tools in at least two of the smartwatches analyzed, because they provide higher sampling rates that can be used by classification algorithms.

In the current work, the frequency response of three smartwatches at different amplitudes has been characterized. The result achieved can be used as a reference for future research work related to the use of accelerometers of wearables devices in different applications such as the prevention of occupational hazards, fall detection or activity or posture detection [39].

The methodology used for the evaluation of the uncertainties for calibration has been presented. The model can be adapted for the evaluation of the uncertainties associated with the measurements of the magnitude of the vibration, which may allow us in the future to use these devices in metrological areas.

It has been identified that the maximum sampling rate and dynamic range to which accelerometers are adjusted within the Android Wear operating system are lower than those at which they could work. Although the design and construction of these sensors allow the use of higher sample rates and dynamic ranges, it is not currently possible to modify these characteristics with the traditional programming methods on the Android platform [83], restricting the operation of the devices of this platform to the factory settings.

Although this kind of wearable device has a reduced cost when compared with the specific measurement equipment, the results show that the sensors have a high potential to be used in different tasks in which it is possible to analyze the acceleration through a built-in accelerometer. With the current configuration, the smartwatches can be used as an option for providing general information about hand–arm vibrations and physical activities related to ORP, but it is necessary to make technical improvements for their use in specific applications such as the assessment of hand–arm vibration, in which limitations in the frequency and amplitude range might be relevant.

Despite the limitations currently imposed by the operating system and the application programming interface, it is possible that solutions will be developed shortly to improve the open issues, such as the battery autonomy of these devices, to avoid reducing the technical characteristics of the sensors. Moreover, if the frequency response curve is known, it is possible to use a reverse filter to improve the frequency linearity in the accelerometer if it is required [34].

## Figures and Tables

**Table 1:** Technical characteristics of the smartwatches and the accelerometers.

	Smartwatch 1	Smartwatch 2	Smartwatch 3
Dimension (mm)	36 × 10 × 51	45.5 × 52.2 × 10.9	45 × 45 × 11.5
Weight (g)	38	66.5	54
Accelerometer amplitude range (standard acceleration due to gravity: *g*)	±2, ±4, ±8, ±16	±2, ±4, ±8, ±16	±2, ±4, ±8, ±16
Accelerometer maximum sample rate (Hz)	2000	4000	1000
Accelerometer maximum sample rate set in Android (Hz)	250	200	50
Analog-to-Digital Converter (ADC) (bit)	12	16	16

**Table 2:** Characterization of the uncertainty sources for calibration.

Variable	Estimator	Density Function	Type	Description
*Ex*	Y	Normal	A	Indication error
*u accel_ref_*	X1	Normal	A	Reference vibrometer RMS acceleration
*u accel_sw_*	X2	Normal	A	Smartwatch RMS acceleration
*u temp_ref_*	X3	Rectangular	B	Reference vibrometer variation due to the temperature
*u temp_sw_*	X4	Rectangular	B	Smartwatch based vibrometer variation due to the temperature
*u res_ref_*	X5	Rectangular	B	Reference vibrometer finite resolution
*u res_sw_*	X6	Rectangular	B	Smartwatch vibrometer finite resolution
*u calib_ref_*	X7	Normal	B	Reference vibrometer uncertainty
*u calib_sw_*	X8	Normal	B	Smartwatch calibration uncertainty
*u cross_sw_*	X9	Rectangular	B	Smartwatch accelerometer cross sensitivity
*u cross_ref_*	X10	Rectangular	B	Reference accelerometer cross sensitivity
*u sw_jitt_*	X11	Normal	B	Smartwatch RMS uncertainty due to the sampling rate variation

**Table 3:** Deviation percentage and relative uncertainty of smartwatch 1 as compared to the reference vibrometer.

		Frequency (Hz)												
Amplitude		6.3	8	10	12.5	16	20	25	31.5	40	50	63	80	100
10 m/s^2^	*ε (%)*	−1.14	1.28	0.63	0.00	0.81	−0.70	−1.15	−1.56	−1.31	−2.81	−4.83	−9.32	−20.64
	*U_rel_*	±3.0	±3.0	±3.0	±3.0	±3.0	±3.0	±3.0	±3.0	±3.0	±3.0	±3.0	±3.0	±3.1
5 m/s^2^	*ε (%)*	1.10	−0.37	−0.20	−0.29	−0.30	−0.65	−1.28	−1.65	−1.12	−2.45	−4.54	−9.13	−20.68
	*U_rel_*	±3.0	±3.0	±3.0	±3.0	±3.0	±3.0	±3.0	±3.0	±3.0	±3.0	±3.0	±3.0	±3.2
1 m/s^2^	*ε (%)*	1.41	0.49	0.25	0.35	−0.45	−0.63	−1.54	−2.51	−1.61	−3.03	−4.84	−9.44	−20.82
	*U_rel_*	±3.1	±3.1	±3.1	±3.1	±3.1	±3.1	±3.1	±3.1	±3.1	±3.1	±3.1	±3.1	±3.8

Values in red: deviation values greater than 6% with respect to the reference.

**Table 4:** Deviation percentage and relative uncertainty of smartwatch 2 as compared to the reference vibrometer.

		Frequency (Hz)											
Amplitude		6.3	8	10	12.5	16	20	25	31.5	40	50	63	80
10 m/s^2^	*ε (%)*	−0.74	1.21	0.73	0.02	0.50	0.24	−0.21	−0.14	1.35	1.56	3.89	8.41
	*U_rel_*	±3.1	±3.0	±3.0	±3.0	±3.0	±3.0	±3.0	±3.0	±3.0	±3.0	±3.0	±3.1
5 m/s^2^	*ε (%)*	0.89	0.23	0.31	−0.49	0.15	−0.10	−0.59	−0.26	1.20	1.29	3.63	7.71
	*U_rel_*	±3.1	±3.0	±3.0	±3.0	±3.0	±3.0	±3.0	±3.0	±3.0	±3.0	±3.0	±3.0
1 m/s^2^	*ε (%)*	2.18	−0.74	−0.57	−0.08	0.17	0.06	−0.85	−0.38	1.50	0.94	3.51	7.06
	*U_rel_*	±3.1	±3.1	±3.1	±3.1	±3.1	±3.1	±3.1	±3.1	±3.1	±3.1	±3.1	±3.1

Values in red: deviation values greater than 6% with respect to the reference.

**Table 5:** Deviation percentage and relative uncertainty of smartwatch 3 as compared to the reference vibrometer.

		Frequency (Hz)						
Amplitude		6.3	8	10	12.5	16	20	25
10 m/s^2^	*ε (%)*	−0.88	0.71	−0.17	0.03	−0.12	−0.29	11.90
	*U_rel_*	±3.1	±3.1	±3.1	±3.2	±3.3	±3.4	±3.6
5 m/s^2^	*ε (%)*	0.74	−0.11	0.06	0.05	−0.04	−1.13	11.68
	*U_rel_*	±3.2	±3.1	±3.1	±3.2	±3.3	±3.4	±3.6
1 m/s^2^	*ε (%)*	−0.42	−1.50	−1.53	−1.84	−1.43	−1.41	7.41
	*U_rel_*	±3.1	±3.1	±3.2	±3.2	±3.3	±3.4	±3.7

Values in red: deviation values greater than 6% with respect to the reference.
